# The effects of exergaming on balance, gait, technology acceptance and flow experience in people with multiple sclerosis: a randomized controlled trial

**DOI:** 10.1186/s13102-015-0001-1

**Published:** 2015-04-17

**Authors:** Jonathan Robinson, John Dixon, Alasdair Macsween, Paul van Schaik, Denis Martin

**Affiliations:** 1 Health and Social Care Institute, Teesside University, Middlesbrough, TS1 3BA UK; 2 School of Social Sciences, Business & Law, Teesside University, Middlesbrough, TS1 3BA UK

**Keywords:** Exergaming, Nintendo Wii™, Balance training, Gait, Multiple sclerosis, Technology acceptance, Flow state experience

## Abstract

**Background:**

Exergaming is a promising new alternative to traditional modes of therapeutic exercise which may be preferable and more effective for people with Multiple Sclerosis (MS). Impaired balance is reported as one of the most disabling aspects of MS. The purposes of this study were to examine the effects of exergaming on: (1) postural sway, (2) gait, (3) technology acceptance and (4) flow experience in people with MS. Secondary outcomes were disability: 12‐item Multiple Sclerosis Walking Scale (MSWS-12) and the World Health Organization Disability Assessment Schedule 2.0 (WHODAS 2.0) questionnaire.

**Methods:**

Fifty-six adults (mean age = 52 years, SD = 5.8; 38 women) with a clinical diagnosis of MS and able to walk 100 meters with or without use of a walking aid were included in this study and randomized into 3 groups. Group 1 received balance training using the Nintendo Wii Fit™ (exergaming) and Group 2 undertook traditional balance training (non-exergaming). Group 3 acted as a control group, receiving no intervention. Exergaming and traditional balance training groups received four weeks of twice weekly balance-orientated exercise. Postural sway was measured using a Kistler™ force platform. Spatiotemporal parameters of gait were measured using a GAITRite™ computerised walkway. Technology acceptance and flow experience were measured using the Unified Theory of Acceptance and Use of Technology and the Flow State Scale questionnaires, respectively.

**Results:**

There were significant improvements in bipedal postural sway in both intervention groups when compared to the control group; and no effects of either intervention on gait. There were no significant differences between the interventions in technology acceptance but on several dimensions of flow experience the Wii Fit™ was superior to traditional balance training. Both interventions showed improvements in disability compared to control.

**Conclusions:**

In terms of the physical effects of exergaming, the Wii Fit™ is comparable to traditional balance training. These findings would support the use of the Wii Fit™ as an effective means of balance and gait training for people with MS, which is both accepted and intrinsically motivating to MS users.

**Trial registration:**

Controlled Trials ISRCTN13924231.

## Background

In Europe and North America the prevalence of Multiple Sclerosis (MS) is estimated at 1:800 people, with an annual incidence of 2–10:100,000 [1]. MS is the most common cause of neurological disability in young adults [1]. MS symptoms are varied and include depression, fatigue, pain, muscle weakness, spasticity, balance and gait problems; all of which lead to reduced physical activity and increased risk of falls [2]. Furthermore, impaired walking ability is estimated to affect upwards of 85% of people with MS [3]. Physical therapy management of people with MS aims to reduce impairment and improve functional ability largely through increasing physical activity [4], participation and independence levels [5]. For people with MS the ability and willingness to increase, and sustain increases in physical activity can be limited by impaired balance, which is considered one of the most disabling symptoms and estimated to affect approximately 75% of people with MS [6].

‘Exergaming’ (exercise using computer-gaming technology) is a relatively new and promising option to encourage physical activity and improve balance [7]. Studies with healthy volunteers have reported general balance improvements in exergaming interventions when combined with traditional exercise [8,9]. Moreover, where exergaming has been the sole intervention, studies have found significant improvements in balance and lower limb muscle strength [10]. Also, in clinical populations exergaming has shown positive results for those with stroke [11], cerebral palsy [12] and Parkinson’s Disease [13] through improvements in balance, gait and function.

Studies on the application of exergaming with the Wii Fit™ to improve balance for people with MS are emerging [14-16]. However, randomized controlled trials on exergaming for MS are required. No previously published trials have used 3 arms to compare exergaming against traditional balance exercises and also a no-intervention control group.

In addition to providing an alternative exercise modality studies have reported psychological benefits with exergaming technology [9,17,18]. Exergaming technology has been shown to lead to greater exercise enjoyment, as well as enhanced exercise concordance [19,20]. Moreover, such interactive gameplay has also demonstrated immersive properties; to the point of reducing perceptions and intensity of effort during physical exercise [21,22]. However, despite the popularity of these systems, research remains limited. There is evidence to suggest that, in non-clinical populations, exergaming is more accepted and engaging than traditional means of exercise [18]. In the current study, the psychological concepts of technology acceptance and flow experience will provide insight into these aspects of therapeutic exercise prescription. Technology acceptance, and therefore intention to use the technology, will be explored using the Unified Theory of Acceptance and Use of Technology (UTAUT) questionnaire for informative technology systems [23], and flow experience, using the Flow State Scale (FSS) questionnaire for use in sport and physical activity [24].

The aim of this study was to investigate the value of exergaming using the Wii Fit™ for people with Multiple Sclerosis. The following experimental non-directional hypotheses were tested: postural sway, gait, technology acceptance, flow experience, and disability in people with MS differ between exergaming, traditional balance training and control.

## Methods

### Design overview

A prospective, randomized controlled three-arm trial design was used. The three arms were exergaming with Wii Fit™, traditional balance training, and no intervention (control group). All testing was carried out by JR who was not blind to participant allocation.

### Setting and participants

Ethical approval was granted by Teesside University and the National Research Ethics Service, permission was given by the South Tees Hospital NHS Foundation Trust R&D Dept. and the trial registered through the International Standard Randomized Controlled Trial Number Register (ISRCTN13924231). This study ran between October 2011 and April 2012. Outcome measures were recorded in the Teesside Centre for Rehabilitation Sciences (TCRS, James Cook University Hospital, Middlesbrough, UK), and interventions undertaken in either the TCRS or the Middlesbrough MS Therapy Centre (non-NHS). Convenience sampling was used to recruit people with MS. Potential participants were referred by a senior MS physiotherapist at James Cook University Hospital or the Middlesbrough MS Therapy centre. Inclusion criteria were: male or female, aged 18–65 years, clinical diagnosis of MS, self-reported ability to walk 100 meters with or without resting with the use of one stick or crutch (equivalent to an Expanded Disability Status Scale [25] score of 6), able to read and comprehend written and spoken English. Exclusion criteria were: acute exacerbation and/or relapse of MS symptoms within the last three months, diagnoses of any other condition affecting the central nervous system, any musculoskeletal injury, or receiving physical therapy.

### Randomization and interventions

Each participant was randomly allocated to one of the groups once written informed consent, demographic information and baseline outcome data had been collected. Participant allocation was stratified by gender and block randomized (blocks of 6) into one of the three groups using an online computer generated sequence created prior to participant recruitment [26]. A follow-up appointment for four weeks was arranged for those allocated to the control group (no intervention). Both intervention groups were then introduced to the allocated programme, undertaking each of the programme-specific exercises, following which they were asked to complete both the UTAUT and FSS questionnaires. Once completed, appointments for four weeks of twice weekly 40–60 minute exercise sessions were arranged at the participant’s convenience [27], at either the hospital or the Middlesbrough MS Therapy Centre. All exercise sessions were completed on a one-to-one basis and under the supervision of the primary researcher (JR, a UK-qualified physiotherapist).

Bespoke exercise programmes were developed for the study. The American College of Sports Medicine [28] states that balance training is one of the least well defined exercise modalities. Given the lack of standardised balance training programmes, combined with the variability of fitness levels in this population, there is no universal exercise training programme for people with MS [29]. As such, balance training can include any activity which stresses balance to elicit adaptations in the control of posture and equilibrium [28]. The Wii Fit™ exercises were categorised using the pre-established Wii Fit™ descriptions [30]; these being *Balance Games*, *Aerobic Games*, and *Muscle Workouts*, all of which are designed to challenge balance to varying degrees. Upon review, the selected Wii Fit™ games were found to mirror common traditional (non-exergaming) balance exercises often prescribed to challenge balance, and consisted of following: Soccer Heading, Ski Slalom, Table Tilt, Tightrope Walk, Rhythm Boxing, Basic Step, and Hula Hoop. These were each completed three times per session. The games Torso Twist and Rowing Squats were completed only once per session. For the Wii Fit™ system, there were two difficulty settings: participants began at *Normal* and increased to *Advanced* upon request. To design a comparable balance training programme these games were assessed in terms of their gross movement patterns and tailored to replicate the actions and demands of the Wii Fit™, based on common stressors of postural control [28], to isolate any effects due to intervention type. For example, the Wii Fit™ game *Tightrope Walk* was mirrored by the traditional balance training group by having the participant walk along a marked straight-line, heel to toe. The selected Wii Fit™ games and comparable traditional balance exercises are listed through Table 1.

**Table 1:** **Intervention exercises for the Wii Fit™ and traditional balance training groups**

Wii Fit™	Traditional balance training	Frequency
**Balance Games**	**Balance Games Equivalent**	
Heading (soccer) (balance board)	Wall Taps (reaching for numbers placed on a wall)	3 times per session
Ski Slalom (balance board)	Standing with feet together; resistance to perturbations	3 times per session
Table Tilt (balance board)	Wobble board (small inflatable)	3 times per session
Tight Rope (balance board)	Straight line walking; heel to toe	3 times per session
**Aerobic Workouts**	**Aerobic Workouts Equivalent**	
Boxing (handheld controllers)	Basic (non-impact) shadow boxing	3 times per session
Step Ups (balance board)	Step ups	3 times per session
Hula Hoop (balance board)	Standing hip rotations	3 times per session
**Muscle Workouts**	**Muscle Workouts Equivalent**	
Torso Twist (balance board)	Torso Twists	1 per session
Rowing Squats (balance board)	Mini squats	1 per session

All exercises were undertaken in standing. Having completed the four week programme the participants repeated the baseline measures (postural sway, gait and questionnaires) within five days of their final exercise session. Questionnaires were completed by the participants independently and without supervision. Those in the control group returned four weeks after baseline to repeat these same measures, with the exception of the intervention-specific questionnaires.

### Outcome measures

The primary outcome measures were postural sway (measured using a force plate), gait (measured using the GAITRite™ walkway), technology acceptance (measured using the UTAUT questionnaire [23]) and flow experience (measured using the FSS questionnaire [24]) recorded at baseline and four weeks after baseline. Secondary outcome measures were self-reported walking ability (measured using the 12‐item Multiple Sclerosis Walking Scale questionnaire [MSWS-12] [31]) and perceived activity ™ and participation restrictions (measured using the 12-item World Health Organization Disability Assessment Schedule 2.0 questionnaire [WHODAS 2.0] [32]) also measured at baseline and four weeks after baseline.

Postural sway data were obtained from a Kistler™ Force plate (Model 9286AA, Kistler, Alton, UK) - W 40 × L 60 × H 3.5 cm - with a sampling rate of 1000 Hz. Postural sway outcomes were the centre of pressure velocity (CoP velocity, mm.sec^−1^), and the range and standard deviation of the excursions in the anterior-posterior (AP) and medio-lateral (ML) directions (AP range, AP SD, ML range, ML SD respectively, all mm) during bipedal and unipedal standing with eyes open. Participants were asked to stand on the Kistler™ force plate with their eyes open (looking at a black 100 mm diameter circle positioned 3 m from the centre of the force plate, positioned at eye level [33,34]) and to remain as relaxed as possible. Parallel bars were placed on either side of participants for safety purposes during all balance testing. An individual trace of foot position was taken for each participant as a means of foot placement standardisation. Participants stood for 30 seconds, three times, with 15 seconds between each, on both feet shoulder-width apart, then, after a two-minute break, three times for 15 seconds on only their dominant (preferred for kicking) leg.

Gait data were obtained using the GAITRite™ walkway system to measure temporal and spatial parameters. The GAITRite™ system is sensitive enough to highlight compromised gait patterns in those with MS who have a very low level of disability and relatively short disease duration [35]. It is a 4.6 m walkway consisting of computerised sensors, arranged in a grid-like pattern to identify footfall contacts [35]. Gait measurements were velocity, Functional Ambulation Profile (FAP), cadence, step length, stride length and heel-to-heel base of support, and were obtained using GAITRite™ software (CIR Systems, Inc., Havertown, PA 19083, USA). All participants walked barefoot along the mat three times, at a self-selected velocity. The mean of these measures were calculated and used for statistical analysis.

Technology acceptance was measured using the UTAUT questionnaire [23]. Each question is measured using a 7-point Likert scale, from 1 (strongly disagree) to 7 (strongly agree). The UTAUT contains 22 items related to the behaviour of exercise, across six subscales: *performance expectancy* (PE; the degree to which the user feels that the system will improve performance), *effort expectancy* (EE; the system’s ease-of-use), *social influence* (SI; the degree to which those important to the user believe they should use the system), *facilitating conditions* (FC; the degree to which the user believes that there is support for using that system, *self-efficacy* (SE; the degree to which a person is confident of using the system) and *behavioural intention* (BI; the degree to which a person has intention to use the system) [23]. The questionnaire was adapted (for exergaming-based specificity) by referring to the specific exercise technology and exercise activity in the current study. Otherwise, the questionnaire remained unchanged. The UTAUT is an accurate predictor of technology acceptance, and in the context of information technology it has been shown to account for 70% of the total variance in prediction of the users’ stated intention to use the technology in the future [23].

Participants‘ flow experience was measured using the FSS questionnaire [24]. The concept of flow experience is linked to high levels of performance [36] and enjoyment [37]. The attainment of flow is described as an intrinsically motivating optimal state, acting to encourage repeat activity [37], and therefore the potential to improve exercise concordance. The principles of flow were adapted by Jackson and Marsh [24] to produce the Flow State Scale (FSS) questionnaire for use in sport and physical activity. Each question is measured using a 5-point Likert scale, ranging from 1 (strongly disagree) to 5 (strongly agree). The FSS contains 36 items, across nine subscales: *autotelic experience* (AE; the activity is intrinsically rewarding), *clear goals* (CG; clear idea of what needs to be accomplished), *challenge-skill balance* (CB; balance between the challenge of the activity and personal skills), *concentration on the task at hand* (CT; complete focused on the task), *paradox of control* (PC; clear feeling of control), *unambiguous feedback* (UF; clear and immediate feedback), *action-awareness merging* (AM; involvement in the task; actions become automatic), *transformation of time* (TT; altered perception of time; either speeding up or down), and *loss of self-consciousness* (LS; no concerns with appearance; focused only the activity). The FSS questionnaire has shown high internal consistency (alpha ranging from 0.79 to 0.86) [24].

Secondary outcomes were the MSWS‐12 [31] and the WHODAS 2.0, [32], completed at baseline and four weeks after baseline for all groups. Previous authors have shown that the MSWS-12 has good internal consistency, high reliability and validity, and good generalizability [38]. The MSWS-12 questionnaire is a disease-specific self‐report measure of walking ability, measured using a 5-point Likert scale, from 1 (Not at all*)* to 5 (Extremely). Items are summed to generate a total score and transformed to a scale with a range of 0 (no disability) to 100 (extreme disability). Higher scores are an indication of a greater impact of MS on walking ability. The WHODAS 2.0 questionnaire is designed to assess the activity limitations and participation restrictions experienced by an individual irrespective of medical diagnosis. For each question the participant is asked to rate the magnitude of the disability from the previous 30 days, selecting to report either: *None* (indicating no difficulty), *Mild*, *Moderate*, *Severe* or *Extreme/cannot*. Higher scores indicate greater disability, and range from 0 (no disability) to 48 (complete disability).

### Exergaming system

The Wii Fit™ (Nintendo Wii™, Nintendo Co Ltd, Minami-ku Kyoto, Japan) uses a central game console, connected to a television. Navigation is via the handheld control pad and physical whole body movements through standing on the Wii Fit™ Balance Board (length 511 mm, width 316 mm and height 53.6 mm). These movements control an avatar in the gaming environment. The games environment and images were displayed on a 37 inch widescreen Plasma screen (Hanspree, Type T73B, Greyenstraat 65, Netherlands).

### Data extraction

The AP and ML CoP excursion variables (mm) were extracted from the force platform using Bioware software, after low-pass filtering of the raw data at 10 Hz. CoP velocity (mm.s^−1^) was calculated using the methods described by Raymakers and Samson [39]. GAITRite™ measures were extracted and relevant parameters saved through the dedicated GAITRite™ software (CIR Systems – version 3.8). Where required the software’s ‘Foot Fall Editor’ was applied to erase the recording of a walking stick or errors in foot placement (errors in the GAITRite™ system recording or footfalls outside the area of measurement) to ensure only participants’ foot falls were analysed.

### Statistical analysis

Data were analysed with Statistical Package for the Social Sciences Version 20 for Windows (SPSS™, Chicago, IL, USA) *as randomized*. Data from all participants who were randomly assigned (following the recording of baseline outcome measures) were analysed using intention-to-treat principles according to their randomized allocation, using complete case analysis [40-42]. To account for any differences between the groups at baseline, separate analyses of covariance (ANCOVA) were conducted for each outcome measure to identify post-intervention differences between the three groups, using the baseline values and age as a covariate, and group and gender as fixed factors. All analyses used a significance level of 0.05. Effect sizes were expressed using Cohen’s *d,* where values of 0.2, 0.5, and 0.8 were interpreted as small, moderate, and large [43], respectively.

### Sample calculation

A power analysis was conducted with the program G*Power Version 3.1.9.2 [44] for a three-group comparison using analysis of variance to detect a large effect (*f* = 0.40) for the postural sway outcome measure and 0.80 power; the results showed the required total sample size was 66. To adjust for a dropout rate of 20%, the total required sample size was 78.

## Results

**Figure 1:**
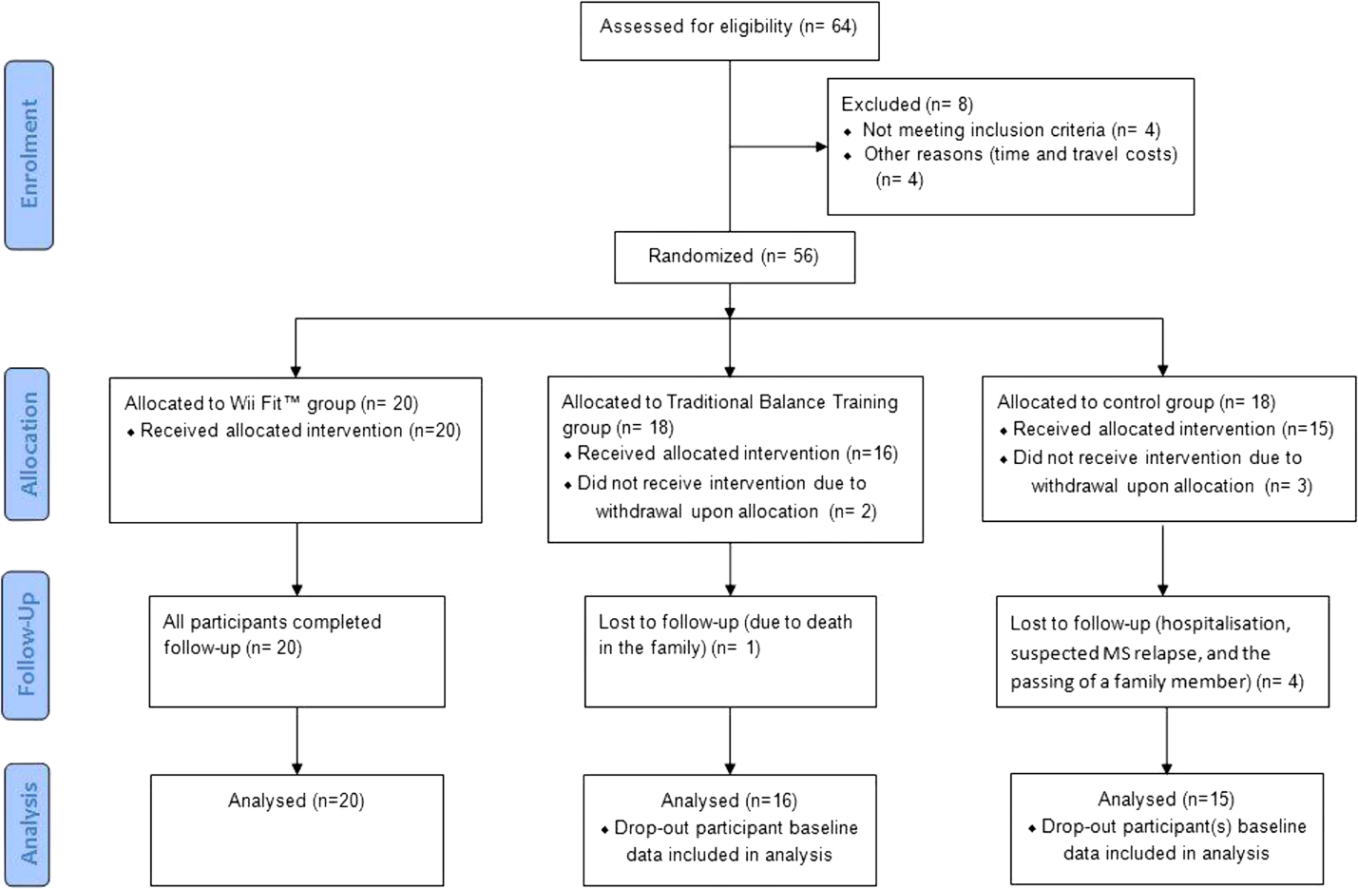
CONSORT flow diagram illustrating a participant entering the study. The final number of participants analysed is based on the principle of complete case analysis and intention-to-treat principles.

Sixty-four participants were screened for eligibility. Eight were excluded due to not meeting the inclusion criteria. Fifty-six (38 females, 18 males; mean age = 52 years, SD = 5.8) were randomly allocated to either: exergaming with the Wii Fit™ (n = 20), traditional balance training (n = 18), or no intervention (control) (n = 18) (see CONSORT Flow Diagram, Figure 1). Five of the randomized participants had withdrawn from the study before start of the intervention. By study completion, an additional five participants had been lost to follow-up due to suspected MS remission, hospitalisation (not related to the study) or family-matters. Therefore, using complete case analysis, statistical analysis was based on n = 20 for Wii Fit™ group; n = 15 for the traditional balance training group; and, n = 11 for the no intervention group. Within the final four weeks of the study no new participants had volunteered to take part, and restricted by time, data collection ended before the stipulated target of 66 participants was achieved. Descriptive statistics for the demographic (Table 2) and (non-adjusted) outcome measures have been provided (Table 3).

**Table 2:** **Participant demographic statistics**

	Wii Fit™	Traditional balance training	Control
	Mean (SD)	Mean (SD)	Mean (SD)
**Age (y)**	52.6 (6.1)	53.9 (6.5)	51.9 (4.7)
**Weight (kg)**	77.7 (17.6)	80.2 (14.5)	84.3 (25.9)
**Height (cm)**	169.4 (10.0)	165.1 (10.1)	156.4 (31.4)
**Gender (f/m)**	14/6	12/7	12/5

**Table 3:** **Descriptive statistics (non-adjusted) for all outcome measures**

			Pre-Programme			Post-programme		
			Wii Fit™	Traditional balance training	Control	Wii Fit™	Traditional balance training	Control
			Mean (SD)	Mean (SD)	Mean (SD)	Mean (SD)	Mean (SD)	Mean (SD)
**Force Plate**	Bipedal	**AP SD** (mm)	9.3 (5.7)	9.5 (4.1)	7.5 (2.8)	8.1 (4.8)	10.5 (9.6)	10.7 (7.0)
		**AP Range** (mm)	45.7 (24.3)	47.3 (18.4)	40.1 (15.1)	37.9 (20.7)	39.0 (17.1)	47.2 (19.1)
		**ML SD** (mm)	5.0 (4.1)	5.3 (3.3)	3.6 (0.8)	4.9 (4.3)	5.0 (3.7)	6.0 (4.4)
		**ML Range** (mm)	29.3 (27.5)	33.8 (21.3)	23.6 (8.5)	26.1 (26.5)	24.3 (14.1)	34.1 (19.5)
		**COP Velocity** (cm.s − 1)	18.5 (4.5)	17.7 (3.4)	19.3 (4.9)	18.0 (4.1)	17.6 (3.7)	20.1 (4.7)
	Unipedal	**AP SD** (mm)	11.1 (3.7)	10.7 (4.1)	10.0 (4.1)	10.0 (4.0)	10.6 (4.5)	12.7 (5.7)
		**AP Range** (mm)	53.9 (20.0)	53.8 (19.8)	51.9 (22.0)	48.3 (19.7)	50.9 (19.4)	63.2 (28.6)
		**ML SD** (mm)	7.4 (4.4)	7.2 (2.6)	6.6 (3.6)	6.7 (3.1)	6.7 (2.5)	7.3 (3.5)
		**ML Range** (mm)	33.7 (19.2)	32.8 (10.7)	32.5 (15.4)	31.9 (14.2)	32.0 (12.7)	39.6 (22.1)
		**COP Velocity** (cm.s − 1)	19.4 (4.0)	18.8 (3.1)	19.7 (4.8)	18.7 (3.4)	18.4 (3.5)	20.1 (4.5)
**GAITRite**		**Velocity** (cm.s − 1)	64.7 (28.9)	76.0 (20.9)	65.5 (24.9)	76.6 (27.1)	80.1 (23.9)	76.6 (36.5)
		**Cadence** (steps/min)	85.0 (20.1)	92.0 (7.1)	86.6 (11.6)	90.1 (16.9)	96.5 (9.5)	90.2 (16.7)
		**Functional Amb. Profile**	69.9 (15.6)	73.1 (15.9)	72.3 (16.0)	78.3 (12.1)	77.4 (16.0)	72.7 (17.7)
		**Step Length** (Left Foot) (cm)	43.2 (12.0)	49.6 (10.9)	45.1 (12.1)	48.6 (11.3)	49.8 (11.5)	49.0 (16.6)
		**Step Length** (Right Foot) (cm)	45.1 (11.4)	48.6 (11.5)	44.4 (13.1)	50.7 (9.5)	48.7 (11.2)	49.2 (15.1)
		**Stride Length** (Left Foot) (cm)	88.1 (22.7)	98.7 (22.6)	89.4 (24.4)	99.4 (20.4)	99.0 (22.6)	98.4 (31.0)
		**Stride Length** (Right Foot) (cm)	88.5 (23.2)	98.2 (21.8)	90.4 (24.6)	100.1 (20.4)	99.6 (22.6)	98.2 (30.6)
		**HH Base Support** (Left Foot) (cm)	14.1 (5.2)	12.1 (4.6)	18.1 (4.1)	14.1 (6.0)	14.0 (4.3)	16.9 (5.7)
		**HH Base Support** (Right Foot) (cm)	14.1 (5.8)	12.6 (4.2)	18.1 (4.1)	14.0 (6.2)	14.0 (4.6)	16.7 (5.5)
**UTAUT**		**Performance Expectancy**	4.9 (1.3)	4.7 (1.4)		6.2 (0.7)	5.5 (1.1)	
		**Effort Expectancy**	4.7 (1.3)	5.2 (1.1)		6.2 (0.8)	5.6 (1.1)	
		**Social Influences**	5.2 (1.1)	5.6 (1.0)		6.1 (0.8)	6.1 (1.0)	
		**Facility Conditions**	4.5 (1.7)	5.6 (0.8)		5.9 (1.2)	6.1 (0.8)	
		**Self-efficacy**	5.1 (1.1)	5.4 (1.3)		6.0 (0.8)	5.9 (1.1)	
		**Behavioural Intention**	5.6 (1.3)	5.9 (0.8)		6.5 (1.1)	6.2 (0.7)	
**FSS**		**Autotelic Experience**	4.2 (0.8)	3.9 (0.8)		4.6 (0.6)	4.1 (0.8)	
		**Clear Goals**	3.5 (0.8)	3.9 (0.8)		4.3 (0.6)	4.0 (0.8)	
		**Challenge-Skill Balance**	3.3 (0.8)	3.7 (0.9)		3.9 (0.5)	4.2 (0.7)	
		**Concentration of Task**	3.9 (0.9)	3.8 (0.7)		4.4 (0.7)	3.9 (0.8)	
		**Paradox of Control**	3.0 (0.9)	3.6 (1.1)		3.8 (0.7)	3.9 (0.8)	
		**Unambiguous Feedback**	3.2 (0.9)	3.7 (1.1)		4.2 (0.7)	3.9 (1.2)	
		**Action-Awareness Merging**	2.8 (0.9)	3.1 (0.8)		3.9 (0.9)	3.3 (0.7)	
		**Transformation of Time**	3.3 (0.7)	2.2 (0.8)		4.1 (0.9)	2.2 (0.9)	
		**Loss of Self-Consciousness**	3.9 (0.9)	4.3 (0.8)		4.3 (0.7)	4.3 (0.9)	
		**MSWS-12 Questionnaire**	64.5 (17.5)	58.4 (16.2)	56.8 (20.7)	59.0 (15.5)	52.3 (16.2)	60.6 (22.4)
		**WHODAS 2.0 Questionnaire**	25.0 (4.7)	25.1 (7.2)	15.6 (7.9)	13.7 (4.7)	14.4 (7.6)	15.8 (8.0)

Note: UTAUT and FSS questionnaires not applicable to control group.

### Postural sway

An ANCOVA revealed a significant main effect of group for BP AP range (*F* [2,40] = 4.1, *p* = 0.02, *d* = 0.91), ML Range (*F* [2,40] = 5.9, *p* = <0.001, *d* = 1.09), and COP velocity (*F* [2,40] = 4.7, *p* = 0.01, *d* = 0.97). Pairwise comparisons identified greater improvement in balance scores in all three of measures in the Wii Fit™ group when compared to control group, and in BP AP and ML range in traditional balance training group when compared to the control group (Table 4). Also, a comparison between the Wii Fit™ and the control group showed that UP COP velocity was found to be approaching significance with a large effect size, in favour of the Wii Fit™ group (*p* = 0.07, effect size *d* = 0.95). However, no significant differences were found between the two intervention groups. There were no statistically significant between-group differences for any of the remaining postural sway measures.

**Table 4:** **Force plate balance measures: Between-group (group effect – ANCOVA) mean diff (95% Confidence Interval [CI]) for force-plate measures: anterior-posterior (AP) and medial-lateral (ML), SD and range (mm), and Centre of Pressure velocity (CoP Velocity) (mm.sec-1) during bipedal and unipedal standing**

		Adjusted post-intervention difference between groups (ANCOVA)								
		Wii Fit™ - control			Traditional balance training - control			Wii Fit™ - Traditional balance training		
		Mean diff. (95% CI)	*P*	*d*	Mean diff. (95% CI)	*P*	*d*	Mean diff. (95% CI)	*P*	*d*
**Bipedal**	**AP SD**	−4.1 (−10.1 to 2.0)	.30	0.65	−2.0 (−8.4 to 4.4)	1	0.32	−2.0 (−7.5 to 3.4)	1	0.32
	**AP Range**	−13.0 (−25.7 to −0.3)	.04*	0.97	−14.2 (−27.7 to −0.6)	.04*	1.05	−1.1 (−10.4 to 12.7)	1	0.09
	**ML SD**	−2.2 (−5.5 to 1.2)	.33	0.62	−2.4 (−5.9 to 1.2)	.31	0.68	0.2 (−2.8 to 3.2)	1	0.06
	**ML Range**	−12.4 (−24.6 to −0.3)	.04*	0.96	−17.7 (−30.7 to −4.7)	.01*	1.37	5.3 (−5.8 to 16.3)	.72	0.41
	**COP Velocity**	−1.4 (−2.6 to −0.3)	.01*	1.15	−1.1 (−2.3 to 0.2)	.12	0.85	−0.4 (−1.5 to 0.7)	1	0.30
**Unipedal**	**AP SD**	−3.3 (−7.7 to 1.0)	.18	0.78	−2.5 (−7.0 to 2.0)	.51	0.59	−0.8 (−4.5 to 2.9)	1	0.19
	**AP Range**	−15.8 (−36.2 to 4.6)	.18	0.79	−13.0 (−34.2 to 8.3)	.40	0.65	−2.8 (−20.2 to 14.6)	1	0.14
	**ML SD**	−1.0 (−3.2 to 1.2)	.75	0.48	−0.9 (−3.2 to 1.4)	1	0.41	−0.2 (−2.0 to 1.7)	1	0.07
	**ML Range**	−8.4 (−20.8 to 3.9)	.29	0.69	−7.6 (−20.5 to 5.3)	.44	0.62	−0.8 (−11.4 to 9.7)	1	0.07
	**COP Velocity**	−1.2 (−2.4 to 0.1)	.07	0.95	−0.8 (−2.2 to 0.5)	.33	0.69	−0.3 (−1.4 to 0.8)	1	0.25

* = significance at the *p* ≤ .05.

### Gait

An ANCOVA revealed no significant between-group post-intervention differences in any of the gait outcome measures (Table 5). However, differences in both Step Length (right foot) and Stride Length (left foot) were found to be approaching significance with moderate effect sizes (*p* = 0.07, effect size: *d* = 0.72 and *p* = .08, effect size *d* = 0.72, respectively) between the Wii Fit™ and traditional balance training group.

**Table 5:** **Gait measures: Between-group (group effect – ANCOVA) mean diff (95% CI)**

	Adjusted post-intervention difference between groups (ANCOVA)								
	Wii Fit™ - control			Traditional balance training - control			Wii Fit™ - traditional balance training		
	Mean diff. (95% CI)	*P*	*d*	Mean diff. (95% CI)	*P*	*d*	Mean diff. (95% CI)	*P*	*d*
**Velocity (cm.s** ^**−1**^ **)**	1.2 (−13.0 to 15.3)	.87	0.07	−5.8 (−21.1 to 9.4)	.44	0.37	7.0 (−5.8 to 19.8)	.27	0.44
**Cadence (steps/min)**	0.9 (−6.5 to 8.4)	.80	0.11	1.6 (−6.4 to 9.6)	.68	0.19	−0.7 (−7.4 to 6.0)	.84	0.08
**Functional Amb. Profile**	7.5 (−1.4 to 16.5)	.13	0.96	4.1 (−5.3 to 13.4)	.84	0.52	3.4 (−4.1 to 11.0)	.78	0.44
**Step Length (Left Foot) (cm)**	2.1 (−4.5 to 8.6)	.53	0.28	−2.9 (−9.9 to 4.2)	.41	0.39	4.9 (−1.1 to 10.9)	.10	0.67
**Step Length (Right Foot) (cm)**	1.2 (−4.6 to 7.1)	.68	0.18	−3.5 (−9.8 to 2.8)	.26	0.53	4.7 (−0.5 to 10.0)	.07	0.72
**Stride Length (Left Foot) (cm)**	3.0 (−9.2 to 15.2)	.62	0.22	−6.8 (−19.9 to 6.3)	.30	0.50	9.8 (−1.2 to 20.9)	.08	0.72
**Stride Length (Right Foot) (cm)**	4.5 (−7.4 to 16.5)	.45	0.33	−4.3 (−17.2 to 8.5)	.50	0.32	8.8 (−2.0 to 19.7)	.11	0.65
**HH Base Support (Left Foot) (cm)**	0.7 (−2.3 to 3.7)	.63	0.23	2.4 (−1.0 to 5.7)	.16	0.75	−1.7 (−4.2 to 0.9)	.20	0.53
**HH Base Support (Right Foot) (cm)**	1.0 (−2.0 to 4.0)	.52	0.30	2.2 (−1.1 to 5.4)	.19	0.68	−1.2 (−3.8 to 1.4)	.35	0.37

### Technology acceptance questionnaire

Baseline scores for the UTAUT were high in both interventions, indicating moderate-to-high acceptance; although, an ANCOVA found no significant between-group post-intervention differences (Table 6). However, differences in two subscales were found to be approaching significance with moderate effect sizes: PE (*p* = 0.08, effect size, *d* = 0.67) and EE (*p* = 0.09, effect size, *d* = 0.66).

**Table 6:** **Unified Theory of Acceptance and Use of Technology questionnaire: Between-subject (group effect – adjusted for baseline differences ANCOVA) mean difference (95% CI)**

	Adjusted post-intervention difference between groups (ANCOVA)		
	Wii Fit™ - traditional balance training		
Subscale	Mean diff. (95% CI)	*P*	*d*
**Performance Expectancy**	0.6 (−0.1 to 1.3)	.08	0.67
**Effort Expectancy**	0.6 (−0.1 to 1.3)	.09	0.66
**Social Influences**	−0.04 (−0.7 to 0.6)	.90	0.05
**Facility Conditions**	0.3 (−0.5 to 1.0)	.46	0.29
**Self-efficacy**	0.2 (−0.4 to 0.7)	.51	0.25
**Behavioural Intention**	0.4 (−0.3 to 1.1)	.29	0.40

### Flow state scale questionnaire

Baseline scores for the FSS questionnaire were high in both intervention groups, indicating moderate-to-high flow experience at the first session. An ANCOVA revealed a significant main effect of group for the subscales CG (*F* [1,30] = 4.0, *p* = 0.05, *d* = 0.74), CT (*F* [1,30] = 5.1, *p* = 0.03, *d* = 0.84), UF (*F* [1,30] = 2.3, *p* = 0.04, *d* = 0.77), AM (*F* [1,30] = 5.1, *p* = 0.03, *d* = 0.84) and TT (*F* [1,30] = 14.3, *p* = <0.001, *d* = 1.37). Pairwise comparisons indicated scores were statistically significantly higher in the Wii Fit™ group for CG, CT, UF, AM and TT (Table 7). No significant differences were found between groups for the remaining subscales. However, the difference in AE was found to be approaching significance, with a *p* value of 0.08 and a moderate effect size (*d* = 0.66).

**Table 7:** **Flow State Scale questionnaire: Between-group (group effect – adjusted for baseline differences ANCOVA) mean differences (95% CI)**

	Adjusted post-intervention difference between groups (ANCOVA)		
	Wii Fit™ - traditional balance training		
Subscale	Mean diff. (95% CI)	*P*	*d*
**Autotelic Experience**	0.4 (0.01 to 0.7)	.08	0.66
**Clear Goals**	0.5 (−0.01 to 0.9)	.05*	0.71
**Challenge-Skill Balance**	−0.2 (−0.5 to 0.2)	.35	0.33
**Concentration of Task**	0.4 (0.04 to 0.8)	.03*	0.78
**Paradox of Control**	0.3 (−0.1 to 0.7)	.17	0.51
**Unambiguous Feedback**	0.5 (0.02 to 1.1)	.04*	0.74
**Action-Awareness Merging**	0.6 (0.06 to 1.2)	.03*	0.78
**Transformation of Time**	1.2 (0.5 to 1.7)	.001*	1.71
**Loss of Self-Consciousness**	0.2 (−0.2 to 0.6)	.23	0.43

* = significance at the *p* ≤ .05.

### WHODAS 2.0 questionnaire

An ANCOVA using the summed WHODAS scores revealed a significant main effect of group (*F* [2,39] = 19.4, *p* = <0.001, *d* = 2.00). Pairwise comparisons identified significantly lower scores in both the Wii Fit™ and traditional balance training groups when compared to the control group (Table 8), with no significant difference between the two intervention groups.

**Table 8:** **WHODAS 2.0 questionnaire: Between-subject (group effect – ANCOVA) mean diff (95% CI)**

	Adjusted post-intervention difference between groups (ANCOVA)								
	Wii Fit™ - control			Traditional balance training - control			Wii Fit™ - traditional balance training		
Item	Mean diff. (95% CI)	*P*	*d*	Mean diff. (95% CI)	*P*	*d*	Mean diff. (95% CI)	*P*	*d*
**WHODAS Score**	−10.2 (−13.5 to −6.8)	<.001*	2.74	−8.7 (−12.2 to −5.2)	<.001*	2.35	−1.5 (−4.1 to 1.1)	.26	0.40

* = significance at the *p* ≤ .05.

### MSWS-12 questionnaire

An ANCOVA identified a moderate-high effect size of group that did not reach significance at the 0.05 level (*F* [2,40] = 2.6, *p* = 0.09, *d* = 0.74). Pairwise comparisons indicated no difference between the Wii Fit™ and the traditional balance group, although there was evidence of lower scores compared to control in the two intervention groups (Table 9).

**Table 9:** **MSWS-12 questionnaire: Between-subject (group effect – ANCOVA) mean diff (95% CI)**

	Adjusted post-intervention difference between groups (ANCOVA)								
	Wii Fit™ - control			Traditional balance training - control			Wii Fit™ - traditional balance training		
Item	Mean diff. (95% CI)	*P*	*d*	Mean diff. (95% CI)	*P*	*d*	Mean diff. (95% CI)	*P*	*d*
**MSWS-12 Scores**	−7.3 (−15.9 to 1.4)	.10	0.65	−9.96 (−18.9 to −0.97)	.03*	0.89	2.7 (−5.1 to 10.5)	.49	0.24

* = significance at the *p* ≤ .05.

## Discussion

The aim of this study was to investigate the effects of exergaming with the Wii Fit™ on postural sway, gait, technology acceptance and flow experience in people with Multiple Sclerosis, in comparison to traditional balance training and no intervention. Overall, we found comparable improvements compared to control in both the intervention groups; as well as significantly higher scores in some flow state subscales in the Wii™ group compared to traditional balance training.

### Postural sway

The findings of the current study with regards to balance improvements are consistent with previous RCTs [15,16]. However, this is the first RCT to compare exergaming with traditional balance training and a control group. As such, our findings provide novel evidence in this area. In general, postural sway improved for participants in both the Wii Fit™ and traditional balance training groups; however, there were no significant post-intervention differences between the intervention groups. This contrasts with Nilsagård et al. [15] who found both Wii Fit™ and control groups improved with no between-group differences, and Brichetto et al. [16] who reported benefit from Wii Fit™ use but not from traditional balance exercises. Our results are a positive finding for exergaming in that they indicate that the Wii Fit™ is comparable to matched traditional balance training as a means of improving postural sway in people with MS, and offers significant improvements over no intervention.

Falls in MS are considered to be linked with increased postural sway [45], and associated with a reduced ability to control movement towards the boundaries of stability and slowed responses to postural disturbances [46]. Using posturography, Porosińska et al. [47] reported that an increased risk of falls is related to an increased postural sway velocity and length of mean sway, which is most pronounced in the medio-lateral direction in those with MS. Conversely, other authors report that increases in postural sway in both directions are associated with increased likelihood of falls [48]. Through the current study, reductions in postural sway measures in both medio-lateral and anterior-posterior directions were found; we can infer this to be a positive finding.

### Gait

Our analysis found no significant post-intervention differences between any of the groups. While we may speculate (with *p* values approaching significance and moderate-to-large effect sizes for Step length and Stride Length) on the potential for differences between the Wii Fit™ and traditional balance training groups, in favour of the Wii Fit™ group, this would require verification in a further study with a higher statistical power than this one.

### Technology acceptance

This is the first study to investigate technology acceptance of exergaming technology through the UTAUT questionnaire in people with MS, providing insight into how people with MS accept and react to exergaming technology compared to traditional interventions. We found high scores at baseline which would suggest high acceptance of both forms of balance training. Our analysis found no significant post-intervention differences between the intervention groups. However, it did suggest (with *p* values approaching significance and moderate effect sizes) the potential for differences for the subscale *performance expectancy* and *effort expectancy*, with higher scores in the Wii Fit™ group. Any such differences would suggest that those in the Wii Fit™ group believed the Wii Fit™ to be more effective in improving balance (performance expectancy), and with a higher degree of ease associated with its use (effort expectancy). The observation on performance expectancy is especially interesting as it is the strongest predictor of intention to use a technology [23]. Again, however, this speculation would require verification in a larger scale study.

### Flow experience

In the current study, we found high scores at baseline for both intervention groups, which suggest that flow state is achieved in new users with first use. We found significantly higher post-intervention scores in the Wii Fit™ group when compared to traditional balance training in five subscales. This suggests that those in the Wii Fit™ group had a higher awareness of what needed to be accomplished (subscale Clear Goals), higher attainable focus (subscale Concentration of Task) and clear immediate feedback (Unambiguous Feedback). In addition, those in the Wii Fit™ group were able to achieve higher levels of involvement in the task whereby movements seemed automatic (subscale Action-awareness Merging), in addition to an altered perception of time (subscale Transformation of Time). These last two subscales may contribute to an important psychological property of exergaming technology, and an advantage over traditional balance training methods – user-distraction [49].

### MSWS-12 and WHODAS 2.0

For the WHODAS 2.0 questionnaire, scores found in this MS sample were distinctly higher than normative data from the general population of a comparable age (normative WHODAS score: 3.2; aged 45–54), as well as those with chronic physical conditions (normative WHODAS score: 5.9; aged 45–54) [50]. We found significantly lower post-intervention scores in both the Wii Fit™ group and traditional balance training groups when compared to the control. Similarly, with the MSWS-12 scores we noted differences from control in both interventions that suggested improvements in both interventions. The lack of statistical significance in the overall ANCOVA and the paired comparison between the Wii Fit™ group and control is, we speculate, most likely to be due to a lack of statistical power. Taken together, these results suggest that both interventions facilitate positive changes to participant health status, function and disability following a four week intervention.

### Clinical relevance

For clinical interpretation of the balance measures with regards to the magnitude of change in postural sway, improvements in the Wii Fit™ group ranged from 3% (BP CoP velocity) to 17% (BP AP Range) having completed a four week intervention. Prosperini et al. [14] reported improvement ranging from 15% to 17% (COP path [mm]) in people with MS completing a 12 week, home-based, Wii™ exercise programme. This offers valuable information for those wishing to employ a similar intervention with regards to exercise duration, with improvements occurring earlier than the findings of Prosperini et al. [14] suggest. A recent study reported that for each 10 mm increase in COP path values there is an 8%-increased risk of being classified as a faller in people with MS [51]. Therefore, our findings indicate the potential for clinically meaningful changes in postural sway which may equate to a reduced risk of falls.

From a clinical perspective, there are clear advantages of an exercise modality which has flow ‘built-in’, since flow achievement is also strongly related to repeat activity and continued use [37,52]. Therefore, the Wii Fit™ has the potential to positively influence exercise concordance, and may offer an accepted alternative to traditional balance training, in which concordance is a problem [53,54]. These findings add an extra dimension to the currently limited information regarding the nature of the players’ experiences [55].

### Limitations of the study

Although this is a larger study compared to most in the specific area of exergaming and MS, it does appear to lack sufficient power to give fully definitive results for some of the comparisons. This research was conducted as part of the completion of a PhD, and, therefore, was restricted by time, geographical area, staffing, and funds. All of the participants volunteered their time and the cost of travel. Larger funded trials would not likely have these restrictions. Where the effect sizes were relatively large the design did show these to be statistically significant as planned. However, as we have indicated, there may have been some instances in which potentially significant differences were not highlighted as such. Where we felt this may have been the case we have emphasised that without verification from a larger analysis any such observation should remain speculative. For practical reasons, neither the participants nor the researcher were blinded and thus the results will contain a degree of bias. The interventions were matched in terms of gross movement pattern akin to traditional balance training exercises; however, these may have differed in terms of physical intensity. A comparable exercise programme to the Wii Fit™, which is matched in terms of intensity, may offer additional insight into the effects of such an intervention in people with MS. Future work should also include a follow-up to assess the duration of any effects, and could investigate the use of the Wii Fit™ without supervision.

## Conclusions

Exergaming was found to be comparable to traditional balance training in terms of its effects on balance and gait, in addition to user-acceptance and self-perceived health related outcomes. However, those using the Wii Fit™ demonstrated significantly higher post-intervention flow state scores in five of the nine subscales, proposing that exergaming may be more intrinsically motivating than traditional balance training. Overall, this would suggest the Wii Fit™ to be an effective and attractive means of balance and gait training, with high acceptance and flow experience in people with MS, which may not only assist in exercise uptake, but also concordance.

## References

[1] Sloka JS, Pryse-Phillips WEM, Stefanelli M (2005). Incidence and prevalence of multiple sclerosis in Newfoundland and Labrador. Can J Neurol Sci..

[2] Matsuda PN, Shumway-Cook A, Ciol MA, Bombardier CH, Kartin DA (2012). Understanding falls in multiple sclerosis: association of mobility status, concerns about falling, and accumulated impairments. Phys Ther..

[3] Scheinberg L, Holland N, Larocca N, Laitin P, Bennett A, Hall H (1980). Multiple sclerosis; earning a living. N Y State J Med..

[4] Stokes M (2004). Physical management in neurological rehabilitation.

[5] Langdon DW, Thompson AJ (1999). Multiple sclerosis: a preliminary study of selected variables affecting rehabilitation outcome. Mult Scler..

[6] Martyn C (2005). Symptoms and signs in the course of disease. McAlpine’s Mult Scler.

[7] Staiano AE, Calvert SL (2011). The promise of exergames as tools to measure physical health. Entertain Comput..

[8] Bateni H (2012). Changes in balance in older adults based on use of physical therapy vs the Wii Fit gaming system: a preliminary study. Physiotherapy..

[9] Brumels KKA, Blasius T, Cortright T, Oumedian D, Solberg B (2008). Comparison of efficacy between traditional and video game based balance programs. Clin Kinesiol..

[10] Nitz JC, Kuys S, Isles R, Fu S (2010). Is the Wii Fit^TM^ a new-generation tool for improving balance, health and well-being? A pilot study. Climacteric..

[11] Kim JH, Jang SH, Kim CS, Jung JH, You JH (2009). Use of virtual reality to enhance balance and ambulation in chronic stroke: a double-blind, randomized controlled study. Am J Phys Med Rehabil..

[12] Deutsch JE, Borbely M, Filler J, Huhn K, Guarrera-Bowlby P (2008). Use of a low-cost, commercially available gaming console (Wii) for rehabilitation of an adolescent with cerebral palsy. Phys Ther..

[13] Esculier J-F, Vaudrin J, Bériault P, Gagnon K, Tremblay LE (2012). Home-based balance training programme using Wii Fit with balance board for Parkinsons’s disease: a pilot study. J Rehabil Med..

[14] Prosperini L, Fortuna D, Giannì C, Leonardi L, Marchetti MR, Pozzilli C (2013). Home-based balance training using the Wii balance board a randomized, crossover pilot study in multiple sclerosis. Neurorehabil Neural Repair..

[15] Nilsagård YE, Forsberg AS, von Koch L (2013). Balance exercise for persons with multiple sclerosis using Wii games: a randomised, controlled multi-centre study. Mult Scler J..

[16] Brichetto G, Spallarossa P, de Carvalho MLL, Battaglia MA (2013). The effect of Nintendo® Wii® on balance in people with multiple sclerosis: a pilot randomized control study. Mult Scler J..

[17] Staiano AE, Calvert SL (2011). Exergames for physical education courses: physical, social, and cognitive benefits. Child Dev Perspect..

[18] Warburton DER, Bredin SSD, Horita LTL, Zbogar D, Scott JM, Esch BTA (2007). The health benefits of interactive video game exercise. Appl Physiol Nutr Metab..

[19] Rhodes RE, Warburton D, Coble J. Effect of interactive video bikes on exercise adherence and social cognitive expectancies in young men: A pilot study. In: Ann Behav Med, vol. Volume 35. 233 Spring St, New York, NY 10013 USA: Springer; 2008. p. S62–2.

[20] Warburton DER, McKenzie DC, Haykowsky MJ, Taylor A, Shoemaker P, Ignaszewski AP (2005). Effectiveness of high-intensity interval training for the rehabilitation of patients with coronary artery disease. Am J Cardiol..

[21] De Bourdeaudhuij I, Crombez G, Deforche B, Vinaimont F, Debode P, Bouckaert J (2002). Effects of distraction on treadmill running time in severely obese children and adolescents. Int J Obes..

[22] Yamashita S, Iwai K, Akimoto T, Sugawara J, Kono I (2006). Effects of music during exercise on RPE, heart rate and the autonomic nervous system. J Sports Med Phys Fitness..

[23] Venkatesh V, Morris M, Davis G, Davis F (2003). User acceptance of information technology: toward a unified view. Manag Inf Syst Q..

[24] Jackson SA, Marsh H (1996). Development and validation of a scale to measure optimal experience: The Flow State Scale. J Sport Exerc Psychol..

[25] Kurtzke JF (1983). Rating neurologic impairment in multiple sclerosis an expanded disability status scale (EDSS). Neurology..

[26] Research Randomizer (Version 4.0) [Computer software] [http://www.randomizer.org/]

[27] Dalgas U, Stenager E, Ingemann-Hansen T (2008). Multiple sclerosis and physical exercise: recommendations for the application of resistance-, endurance- and combined training. Mult Scler..

[28] American College of Sports Medicine (2010). ACSM’s resource manual for guidelines for exercise testing and prescription.

[29] American College of Sports Medicine (2009). ACSM’s exercise management for persons with chronic diseases and disabilities.

[30] Wii Fit^TM^ Plus, Balance Games [http://www.wiifit.com/training/balance-games.html]

[31] Hobart JC, Riazi A, Lamping DL, Fitzpatrick R, Thompson AJ (2003). Measuring the impact of MS on walking ability The 12-Item MS Walking Scale (MSWS-12). Neurology..

[32] WHO | WHO Disability Assessment Schedule 2.0 [http://www.who.int/classifications/icf/whodasii/en/]

[33] Schulmann DL, Godfrey B, Fisher AG (1987). Effect of eye movements on dynamic equilibrium. Phys Ther..

[34] Rome K, Brown CL (2004). Randomized clinical trial into the impact of rigid foot orthoses on balance parameters in excessively pronated feet. Clin Rehabil..

[35] Givon U, Zeilig G, Achiron A (2009). Gait analysis in multiple sclerosis: characterization of temporal–spatial parameters using GAITRite functional ambulation system. Gait Posture..

[36] Jackson S, Eklund R (2002). Assessing flow in physical activity: the flow state scale-2 and dispositional flow scale-2. J Sport Exerc Psychol..

[37] Csikszentmihalyi M (1990). Flow: the psychology of optimal experience.

[38] Motl RW, Snook EM (2008). Confirmation and extension of the validity of the Multiple Sclerosis Walking Scale-12 (MSWS-12). J Neurol Sci..

[39] Raymakers JA, Samson MM, Verhaar HJJ (2005). The assessment of body sway and the choice of the stability parameter(s). Gait Posture..

[40] Heritier SR, Gebski VJ, Keech AC (2003). Inclusion of patients in clinical trial analysis: the intention-to-treat principle. Med J Aust..

[41] Montedori A, Bonacini MI, Casazza G, Luchetta ML, Duca P, Cozzolino F (2011). Modified versus standard intention-to-treat reporting: Are there differences in methodological quality, sponsorship, and findings in randomized trials? A cross-sectional study. Trials..

[42] Allison PD. Handling missing data by maximum likelihood. In SAS® Glob Forum 2012 Conf SAS Inst (Paper 312–2012). Cary, NC; 2012.

[43] Cohen J (1988). Statistical power analysis for the behavioral sciences.

[44] Faul F, Erdfelder E, Lang A-G, Buchner A (2007). G*Power 3: A flexible statistical power analysis program for the social, behavioral, and biomedical sciences. Behav Res Methods..

[45] Shafizadeh M, Abolfazli R, Platt GK (2012). Effect of visual force biofeedback on balance control in people with Multiple Sclerosis-a Pilot Quasi-experimental study. J Phys Ther..

[46] Cameron MMH, Lord S (2010). Postural control in multiple sclerosis: implications for fall prevention. Curr Neurol Neurosci Rep..

[47] Porosińska A, Pierzchała K, Mentel M, Karpe J (2010). Evaluation of postural balance control in patients with multiple sclerosis–effect of different sensory conditions and arithmetic task execution. A pilot study. Neurol Neurochir Pol.

[48] Sosnoff J, Socie M, Boes M, Sandroff B (2011). Mobility, balance and falls in persons with multiple sclerosis. PLoS One..

[49] Kato PM (2010). Video games in health care: closing the gap. Rev Gen Psychol..

[50] Andrews G, Kemp A, Sunderland M, Von Korff M, Ustun TB (2009). Normative data for the 12 item WHO Disability Assessment Schedule 2.0. PLoS One..

[51] Prosperini L, Pozzilli C (2013). The clinical relevance of force platform measures in multiple sclerosis: a review. Mult Scler Int..

[52] Jones MG. Creating electronic learning environments: games, flow, and the user interface. In: Proc Sel Res Dev Present Natl Conv Assoc Educ Commun Technol. St. Louis, MO: ERIC; 1998.

[53] Middleton A (2004). Chronic low back pain: patient compliance with physiotherapy advice and exercise, perceived barriers and motivation. Phys Ther Rev..

[54] Campbell R, Evans M, Tucker M, Quilty B, Dieppe P, Donovan JL (2001). Why don’t patients do their exercises? Understanding non-compliance with physiotherapy in patients with osteoarthritis of the knee. J Epidemiol Community Health..

[55] Thin AG, Hansen L, McEachen D (2011). Flow experience and mood states while playing body movement-controlled video games. Games Cult..

